# High-throughput phenotypic screen identifies a new family of potent anti-amoebic compounds

**DOI:** 10.1371/journal.pone.0280232

**Published:** 2023-05-09

**Authors:** Conall Sauvey, Ittipat Meewan, Gretchen Ehrenkaufer, Jonathan Blevitt, Paul Jackson, Ruben Abagyan

**Affiliations:** 1 Center for Discovery and Innovation in Parasitic Diseases, Skaggs School for Pharmacy and Pharmaceutical Sciences, University of California—San Diego, La Jolla, California, United States of America; 2 Institute of Molecular Biosciences, Mahidol University, Nakhon Pathom, Thailand; 3 Division of Infectious Diseases, Department of Internal Medicine, Stanford University School of Medicine, Stanford, California, United States of America; 4 Janssen Research and Development, LLC, Ja Jolla, California, United States of America; Centro de Investigación y de Estudios Avanzados del I.P.N., MEXICO

## Abstract

*Entamoeba histolytica* is a disease-causing parasitic amoeba which affects an estimated 50 million people worldwide, particularly in socioeconomically vulnerable populations experiencing water sanitation issues. Infection with *E*. *histolytica* is referred to as amoebiasis, and can cause symptoms such as colitis, dysentery, and even death in extreme cases. Drugs exist that are capable of killing this parasite, but they are hampered by downsides such as significant adverse effects at therapeutic concentrations, issues with patient compliance, the need for additional drugs to kill the transmissible cyst stage, and potential development of resistance. Past screens of small and medium sized chemical libraries have yielded anti-amoebic candidates, thus rendering high-throughput screening a promising direction for new drug discovery in this area. In this study, we screened a curated 81,664 compound library from Janssen pharmaceuticals against *E*. *histolytica* trophozoites *in vitro*, and from it identified a highly potent new inhibitor compound. The best compound in this series, JNJ001, showed excellent inhibition activity against *E*. *histolytica* trophozoites with EC_50_ values at 0.29 μM, which is better than the current approved treatment, metronidazole. Further experimentation confirmed the activity of this compound, as well as that of several structurally related compounds, originating from both the Janssen Jump-stARter library, and from chemical vendors, thus highlighting a new structure-activity relationship (SAR). In addition, we confirmed that the compound inhibited *E*. *histolytica* survival as rapidly as the current standard of care and inhibited transmissible cysts of the related model organism *Entamoeba invadens*. Together these results constitute the discovery of a novel class of chemicals with favorable *in vitro* pharmacological properties. The discovery may lead to an improved therapy against this parasite and in all of its life stages.

## Introduction

*Entamoeba histolytica* is a parasitic protozoan amoeba that infects the human intestinal tract and causes intestinal disease with symptoms include stomach cramping, abdominal pain, and diarrheal disease amoebiasis, also known as amoebic colitis [1, 2]. In some cases, the localized diseases were disseminated to more severe diseases such as liver abscess, pneumonia, purulent pericarditis, and cerebral amoebiasis [3, 4]. It is estimated to infect around 50 million people globally at any given time, resulting in approximately 50,000 to 70,000 deaths annually, and as such represents a significant problem from a global health perspective [5–7]. It exists in a two-stage life cycle consisting of an environmental-resistant, infective cyst stage, and a mobile, invasive trophozoite stage. It is transmitted in a characteristic fecal-oral route, where cysts can be ingested from contaminated water or food. Once ingested, cysts pass through to the host intestinal tract where they release trophozoites. Trophozoites feed and multiply, and under certain conditions invade and infect the surrounding host tissues. They can further re-form into cysts which are passed in the host’s feces, and potentially onwards to other hosts [6]. Due to this mode of infection, amoebiasis is most widespread in places where fecal contamination of water or food is likely, such as those where water sanitation is insufficient or non-existent. Thus, it most heavily affects and damages populations that are already socioeconomically vulnerable [7–9]. Symptomatic amoebiasis occurs when trophozoites attack the intestinal lining, causing ulceration, and invade the surrounding tissues [10]. Symptoms usually include long-lasting diarrhea progressing to dysentery, as well as generalized abdominal tenderness and fever [1]. In extreme cases infection can spread from the intestinal region to others such as the liver, lungs, or brain [6, 11]. All of these are significantly more dangerous and more likely to result in mortality.

The current standard of care for amoebiasis is the nitroimidazole drug metronidazole, commonly known under the brand name “Flagyl.” Metronidazole has seen widespread use against both amoebiasis and other protozoan parasitic diseases since its discovery in the mid-20th century [12]. Despite its successes however, several critical issues exist with metronidazole which render necessary the continued search for new treatment options. One of these issues is the significant adverse effects that often accompany metronidazole treatment. Several of these adverse effects are similar to the symptoms of amoebiasis itself, such as diarrhea and fever, and can exacerbate the already difficult experience for the patient to the point of intolerability [13]. In fact, a study in Rwanda has found these effects to be associated with patient non-compliance with the course of metronidazole treatment, as well as worse clinical outcomes [14]. Another issue with metronidazole is its inability to kill the transmissible cyst stage of *E*. *histolytica*, necessitating follow up treatment with an additional drug such as paromomycin in order to prevent disease spread [15]. This complication and extension of the overall course of treatment could possibly also reduce the likelihood of full patient compliance with the treatment regimen. Beyond these issues, a final concern is the possibility of emergent resistance to metronidazole therapy. While this has yet to be reported in the field, resistant strains of *E*. *histolytica* are routinely generated in laboratory settings [16–18].

Given these gaps in the effectiveness of metronidazole therapy for amoebiasis, the search for new and additional compounds capable of inhibiting this parasite is ongoing [19]. In particular, several screening efforts of various sizes have been undertaken, and have produced multiple compounds of interest [20, 21]. Furthermore, FDA-approved drug auranofin have been identified as an inhibitor of *E*. *histolytica* trophozoite growth from a screen of the 910-member Iconix drug library [20]. More recently, the study on a low-throughput targeted screen of antineoplastic kinase inhibitor drugs, identifying multiple highly-potent *E*. *histolytica* inhibitors including the cancer chemotherapy drug ponatinib [22]. Interestingly, another, much larger screen of the 11,948-member ReFRAME library conducted in parallel by authors of this study also identified ponatinib among its potent hits [21].

Given the success of these past screen-based studies, in this study we expanded upon their approach by conducting a high-throughput, semi-automated phenotypic screen of a large chemical library in collaboration with Janssen Pharmaceuticals, Inc. against *E*. *histolytica*, *in vitro*. This library, called the Jump-stARter library, consists of 81,664 small molecules selected for their favorable chemical properties for drug development purposes, as well as their structural diversity. The library was designed with these features in order to “jump-start” novel drug discovery efforts and allow for the identification of structures and structural features that are active in a specified context [23–28]. From the screen of this library, we found a highly active compound against *E*. *histolytica* trophozoites. Further investigation showed similar activity among several structural-related compounds included in the library, as well as several additional such compounds purchased from commercial vendors. Of these compounds, we found nearly all of them to be non-toxic to a cultured human cell line. We also identified activity of the initial compound against cysts of the related model organism *Entamoeba invadens*, and found it to inhibit *E*. *histolytica* at the same rate as metronidazole. Together these results show this new compound to be a promising candidate for drug development against amoebiasis.

## Materials and methods

### Compound library and chemical properties prediction

The chemical compound library screened against *E*. *histolytica* trophozoites was obtained in collaboration with Janssen Pharmaceuticals. The library, referred to as the “Jump-stARter” library, contains a diverse collection of 81,664 drug-like small molecules intended for maximum potential efficacy in collaboration drug-discovery projects with external research groups [23–26, 28]. The Jump-stARter library was originally selected from millions of proprietary compounds by Janssen Pharmaceuticals medicinal chemists using “drug-likeness,” structural diversity, and favorable physical properties as criteria [27, 28]. For this study the library was spotted into black, clear-bottom 1536-well plates (Greiner) at 50nL per well in the Janssen compound logistics facility in Beerse, Belgium, and shipped directly to the University of California—San Diego. Top compounds in this study were predicted the additional key pharmacokinetic property including solubility, lipophilicity and ICM ToxScore by the method implemented in ICM-Pro v3.9 [29]. ICM ToxScore of a compound was calculated based on the present of substructure or functional group that were identified as toxic [30, 31].

### *Entamoeba* cell culture

*E*. *histolytica* strain HM-1:IMSS trophozoites were maintained in 50 ml culture flasks (Greiner Bio-One) containing TYI-S-33 media, 10% (v/v) heat-inactivated adult bovine serum (Sigma), 1% (v/v) MEM Vitamin Solution (Gibco), supplemented with penicillin (100 U/mL) and streptomycin (100 μg/mL) (Omega Scientific) [20]. *E*. *invadens* strain IP-1 were cultured in LYI-S-2 at 25°C [32, 33].

### High-throughput phenotypic screen

Metronidazole and DMSO controls were added to the pre-spotted 1536-well Jump-stARter library plates using a Multidrop Combi reagent dispenser (Thermo Scientific), resulting in final assay concentrations of 25 μM and 0.5% (v/v) respectively. Cultured *E*. *histolytica* trophozoites were then seeded into the same plates at a density of 400 cells per well, also using a Multidrop Combi reagent dispenser, resulting in a final test compound concentration of 25 μM with the total volume of 2 μL. The plates were sealed into GasPak EZ (Becton-Dickinson) bags and incubated at 37°C for 48hr. 2 μL of CellTiter-Glo luminescence-based cell viability assay reagent was added using a Multidrop Combi reagent dispenser, and plates were incubated in the dark for 10 minutes. Luminescence was measured using an EnVision plate reader (Perkin Elmer). Percent inhibition values for each well were calculated and plotted using Microsoft Excel. Outlying values and plates resulting from observed technical malfunctions such as clogging or bubbling during liquid handling were excluded from the final analysis.

### 384-well dose-response cell viability assay

Compounds at 5 mM were transferred from a stock plate to black, clear-bottom 384-well plates (brand) and each diluted in DMSO in a 7-point series by a factor of 2. Diluted compounds were then transferred in triplicate into black, clear-bottom 384-well assay plates using an Acoustic Transfer System (EDC Biosystems). Final compound concentrations ranged from 25 μM to 0.39 μM. Cultured *E*. *histolytica* trophozoites were added at a density of 1000 cells per well, plates were sealed into GasPak EZ bags with the total volume of 100 μl and incubated at 37°C for 48hr. 20 μl of CellTiter-Glo reagent was added, and luminescence values were measured, and percent inhibition was calculated for each well as described previously.

### 96-well dose-response EC_50_ determination assay

Following the method that previously published cultured *E*. *histolytica* trophozoites were seeded into white, solid-bottom 96-well plates (Greiner) at a density of 5000 cells per well [20]. Selected compounds were diluted in DMSO in 8-point series by a factor of 2 and added to wells in the total volume of 100 μl. The final concentration of metronidazole and DMSO controls were 10 μM and 0.5% (v/v), respectively in the total volume of 100 μl each well. Plates were sealed into GasPak EZ bags and incubated at 37°C for 48hr. Then, 50 μl of CellTiter-Glo reagent was added, and luminescence values were measured, and percent inhibition was calculated for each well as described previously. All assays were performed in triplicate. EC_50_ values were calculated, and dose-response curves were plotted using GraphPad Prism software.

### Human cell toxicity assay

HEK293 cells were cultivated in 75cm2 flasks (Corning) containing Dulbecco’s modified eagle medium (DMEM) (Gibco) supplemented with 10% (v/v) fetal bovine serum (FBS) (Gibco) and antibiotic-antimycotic solution (Sigma-Aldritch). Cells were detached by trypsinization, collected, and seeded into (plates) at a density of 5000 cells per well. Test compounds were diluted in DMSO in a 6-point series by a factor of 2 and added to assay wells in quadruplicate alongside 0.5% (v/v) DMSO and media-only controls. Final assay concentrations ranged from 25 μM to 0.78 μM. Assay plates were incubated for 48 hours, then CellTiter-glo reagent was added. Assay plates were incubated in the dark for 10 minutes, then luminescence values were read on an EnVision plate reader. Percent inhibition values for each replicate were calculated from luminescence data using Microsoft Excel, and LD_50_ values were calculated, and dose-response curves plotted using GraphPad Prism.

### Time course assay

Cultured *E*. *histolytica* trophozoites were plated in 96-well plates at densities of 5000 cells per well. Compounds of interest were serially diluted by a factor of 2 in DMSO to a total of 8 points and added to wells in each replicate plate in triplicate along with 0.5% (v/v) DMSO and 10 μM metronidazole controls. The final in-well concentrations of the compounds of interest ranged from 25 μM to 0.195 μM. The replicate plates were individually incubated for either 6, 12, 24, 36, or 48 hours, following which CellTiter-glo reagent was added. Luminescence was measured, EC_50_ values were determined, and dose-response curves were plotted as described previously.

### Cyst viability assay

Mature cyst viability assay was performed as described previously, using a transgenic *E*. *invadens* line stably expressing luciferase (CK-luc) [19, 25, 34, 35]. Parasites were induced to encyst by incubation in encystation media (47% LG) ^26^. After 72 h, parasites were washed once in distilled water and incubated at 25°C for 4–5 h in water to lyse trophozoites. Purified cysts were pelleted, counted to ensure equal cyst numbers using a hemocytometer, and resuspended in encystation media at a concentration of 1.5x10^5^ cells per ml. One ml suspension per replicate was transferred to glass tubes containing encystation media and drug or DMSO, then incubated at 25°C for 72 h. On the day of the assay, cysts were pelleted and treated once more with distilled water for 5 h to lyse any trophozoites that had emerged during treatment. Purified cysts were then resuspended in 75 μl Cell Lysis buffer (Promega) and sonicated for 2x10 seconds to break the cyst wall. 35 μl of each sample was then transferred into white, solid-bottom 96-well plates (Greiner). The equal volume of Promega luciferase assay reagent (35 μl) was added to the volume of lysate and mixed for 2 minutes on the shaker. The luminescence signals were recorded at room temperature and not normalized to protein content. Effect of the drug was calculated by comparison to DMSO control, after subtraction of background signal. Significance of drug effects was calculated using a one-tailed T-test.

## Results

### High-throughput screen of the Jump-stARter library against *E*. *histolytica* trophozoites *in vitro*

**Fig 1:**
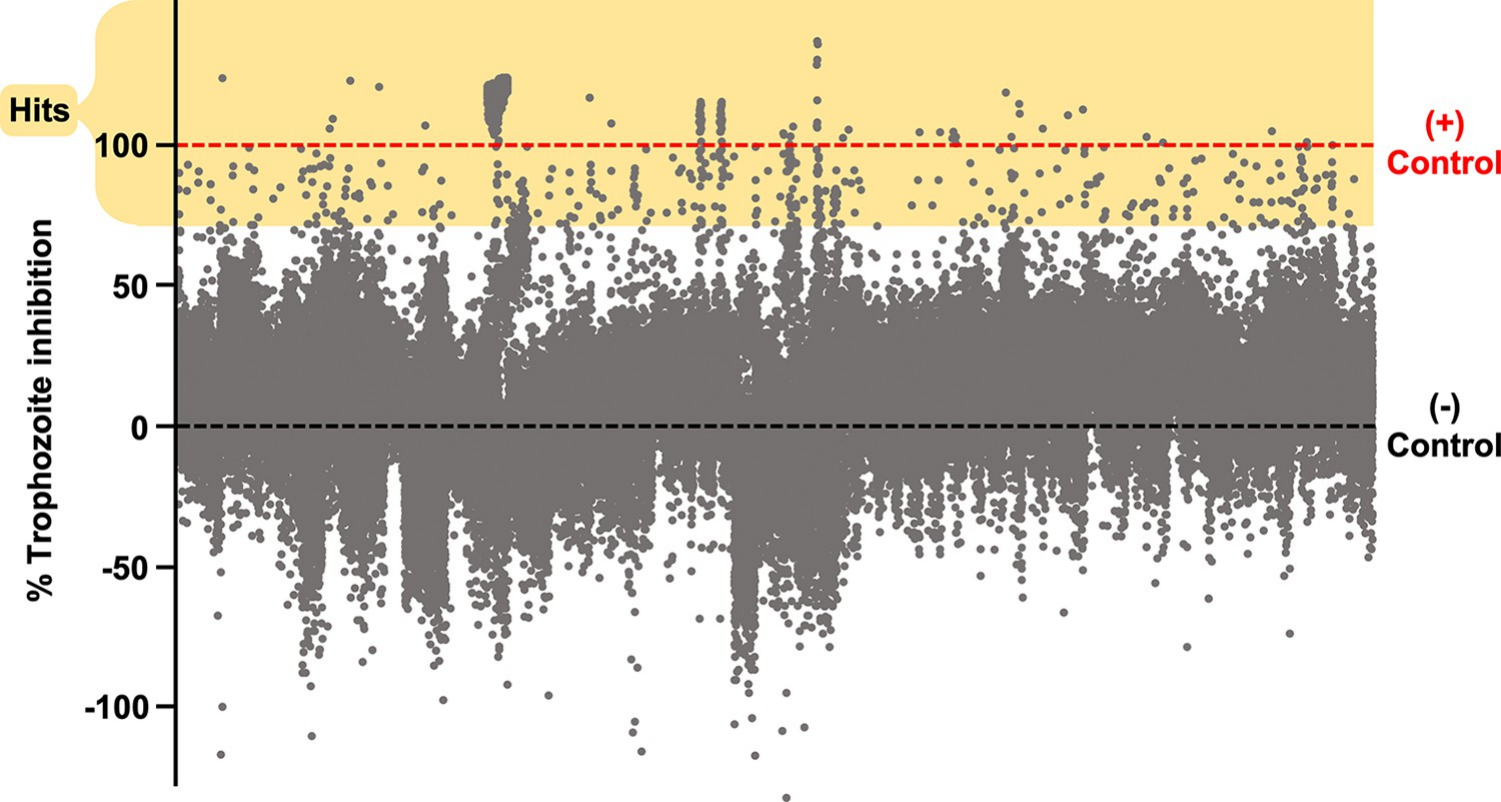
High-throughput screening results of 81,664 small molecules against *E*. *histolytica*. Scatterplot displays percent inhibition values of Jump-stARter library compounds against *E*. *histolytica* trophozoites calculated relative to positive and negative controls. Grey dots represent individual compound values. Red dashed line represents 100% (average of 10 μM Metronidazole, positive controls) and black dashed line represents 0% (average of 0.5% (v/v) DMSO, negative control) percent inhibition values. Yellow box encloses compounds with a percent inhibition value greater than 70%, which were marked as hits and selected for further investigation.

In order to identify new inhibitors of the human parasite *E*. *histolytica* we established a collaboration with scientists from Janssen Pharmaceuticals, inc., and from them, obtained a copy of their Jump-stARter chemical library. This library consists of 81,664 small molecules selected by Janssen medicinal chemists for their chemical diversity and optimal physical properties for drug discovery and development efforts [23, 24, 27, 28]. We screened the Jump-stARter library against *E*. *histolytica* trophozoites *in vitro* using a semi-automated, high-throughput methodology. Previous screens using this organism have been accomplished using 96-well or 384-well plate formats, but due to the large size of the Jump-stARter library, we developed and utilized a novel 1536-well-plate-based methodology. All compounds in the library were tested at 25 μM, and the viability of the parasite cells after incubation for 48 hours was measured with the luciferase-based CellTiter-glo assay. 297 compounds achieved greater than 70% inhibition values and were thus designated as ‘hits’ and selected for further investigation (Fig 1).

### Dose-response screen of top candidate molecules to determine potency

**Fig 2:**
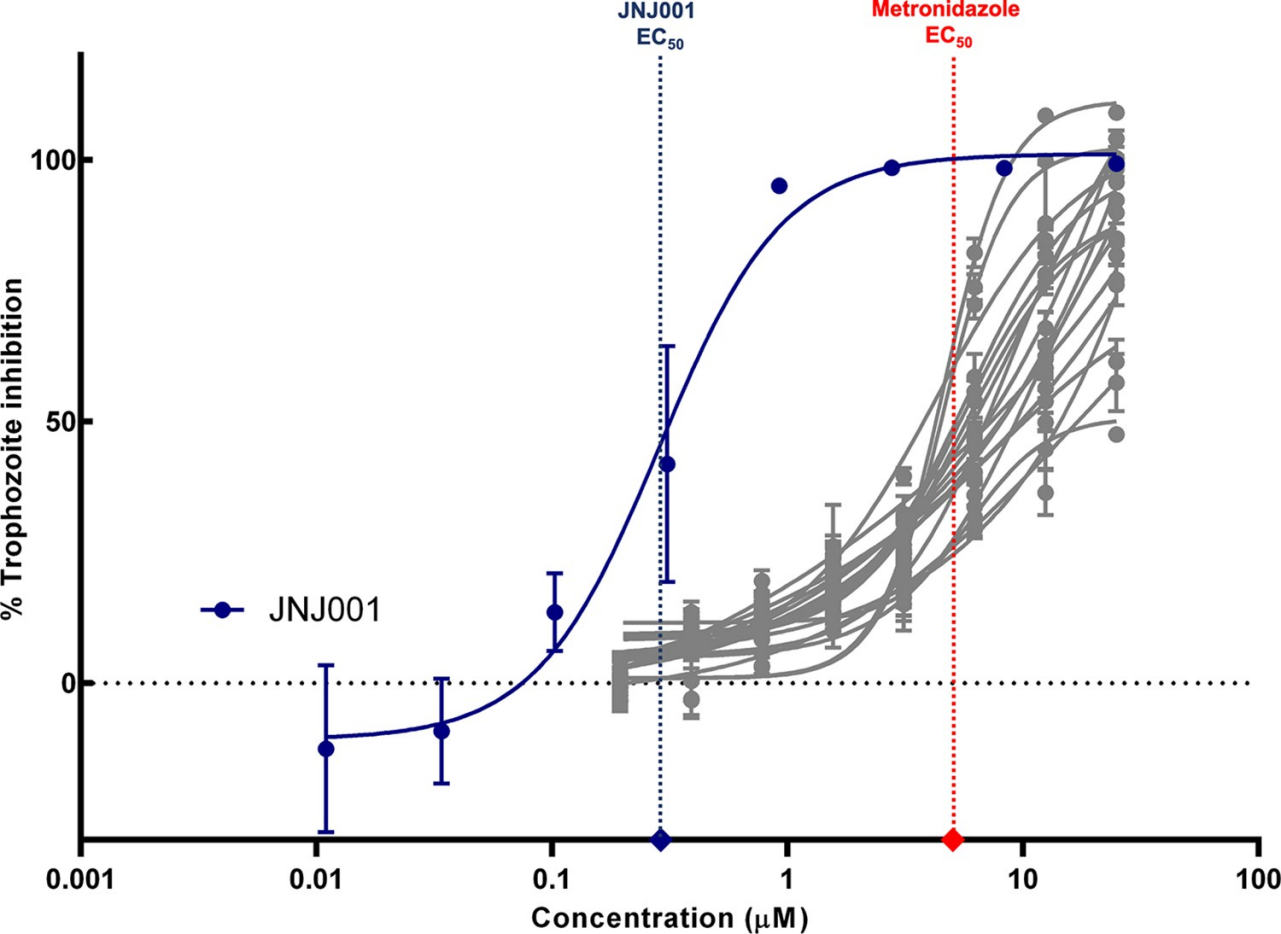
EC_50_ determination assay identifies a highly potent inhibitor of *E*. *histolytica*. Dose-response curves show percent inhibition of *E*. *histolytica* trophozoites compared to compound concentration. Trophozoite cell viability was measured after 48 hours of exposure to test compounds, and percent inhibition for each compound was calculated relative to controls. Compound JNJ001 (displayed in dark navy blue) was assayed over a broader range of concentrations compared to other compounds (displayed in grey) due to previous results indicating high potency in lower concentration ranges. Vertical dotted lines represent EC_50_ values of compound JNJ001 (navy blue) and metronidazole (red). Horizontal dotted line represents 0% inhibition level. Error bars represent standard deviation calculated from three measurements of each concentration.

Of the 297 hits from the high-throughput screen, 128 were available to be ordered in additional amounts from Janssen pharmaceuticals. In order to determine their potency, we ordered stock solutions of these compounds and conducted a medium-throughput dose-response screen against *E*. *histolytica* trophozoites *in vitro*. The compounds were diluted into triplicate 7-point dose-response curves ranging in final assay concentration from 25 μM to 0.39 μM in a 384-well plate format. Parasite cells were incubated with the compounds, and their viability measured using CellTiter-glo (S2 Dataset). 19 compounds which met the minimum criteria of either producing high levels of parasite inhibition across several points of concentration, or producing a varying range of inhibitions across the different concentrations were selected for further in-depth investigation. Compounds which achieved low or moderate levels of inhibition consistently across all points of concentration were excluded. In order to determine their EC_50_ values, the 19 compounds were tested against *E*. *histolytica* trophozoites in triplicate 8-point dose response curves in 96-well plates (Fig 2). From these, a compound designated hereafter as JNJ001 achieved the remarkably low EC_50_ value of 0.29 μM, with the other compounds achieving values ranging from 4.30 μM to 15.72 μM (Table 1). EC_50_ calculated from JNJ014 to JNJ019 were higher than 25 μM, therefore, those six compounds were identified as no effect. These results revealed compound JNJ001 as the top hit, with high inhibitory potency against *E*. *histolytica* trophozoites.

**Table 1:** EC_50_ values of top hits from high-throughput screen.

Compound ID:	EC_50_ (μM)
Metronidazole	2−5^22^
JNJ001	0.29
JNJ002	4.30
JNJ003	4.59
JNJ004	4.59
JNJ005	5.45
JNJ006	5.78
JNJ007	5.78
JNJ008	6.09
JNJ009	6.39
JNJ010	8.37
JNJ011	10.26
JNJ012	10.92
JNJ013	15.72
JNJ014	No effect
JNJ015	No effect
JNJ016	No effect
JNJ017	No effect
JNJ018	No effect
JNJ019	No effect

EC_50_ values determined from dose-response assay against *E*. *histolytica* trophozoites *in vitro*.

### Cell counting assay confirms inhibitory activity against *E*. *histolytica* trophozoites

**Fig 3:**
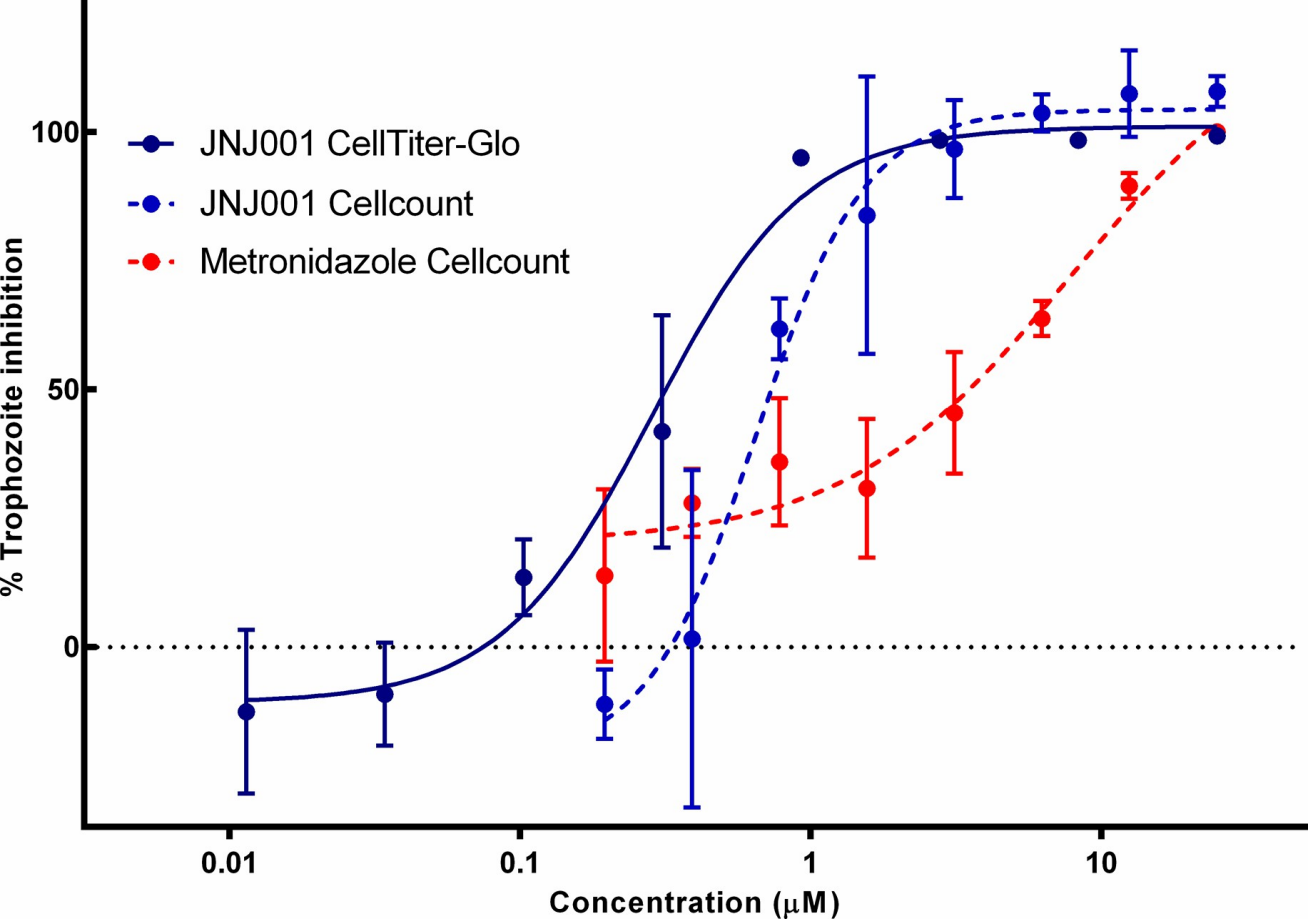
Cell counting assay confirms anti-amoebic activity of compound JNJ001. Dose-response curves show percent inhibition of *E*. *histolytica* trophozoites compared to compound concentration. Trophozoite cell viability or survival was measured after 48 hours of exposure to test compounds, and percent inhibition for each compound was calculated relative to controls. Dashed lines represent parasite survival measured by cell counting. Solid line represents parasite cell viability measured by a luminescence-based assay. Error bars represent standard deviation calculated from three individual measurements for each concentration.

In order to confirm the activity of compound JNJ001 against *E*. *histolytica*, it was once again tested in an 8-point dose-response curve in triplicate in a 96-well plate, along with metronidazole as a control compound. In this assay however, survival of the parasites was directly determined by cell counting using a hemocytometer. The results of this counting assay were match closely when plotted alongside to the Luciferase-based dose-response curve results and the EC_50_ calculated from this assay was 0.58 μM which was comparable to EC_50_ 0.29 μM from Luciferase-based assay (Fig 3). This comparison supports the accuracy of the luciferase-based assay results. The therapeutic index (TI) for JNJ001 (EC_50_ values determined from dose-response assay against *E*. *histolytica* trophozoites divided by approximated LD_50_ the toxicity assay against human HEK293 cells) was over 86. The overall results indicate that compound JNJ001 does in fact act as a potent inhibitor of *E*. *histolytic*a trophozoites.

### Human cell toxicity assay

**Fig 4:**
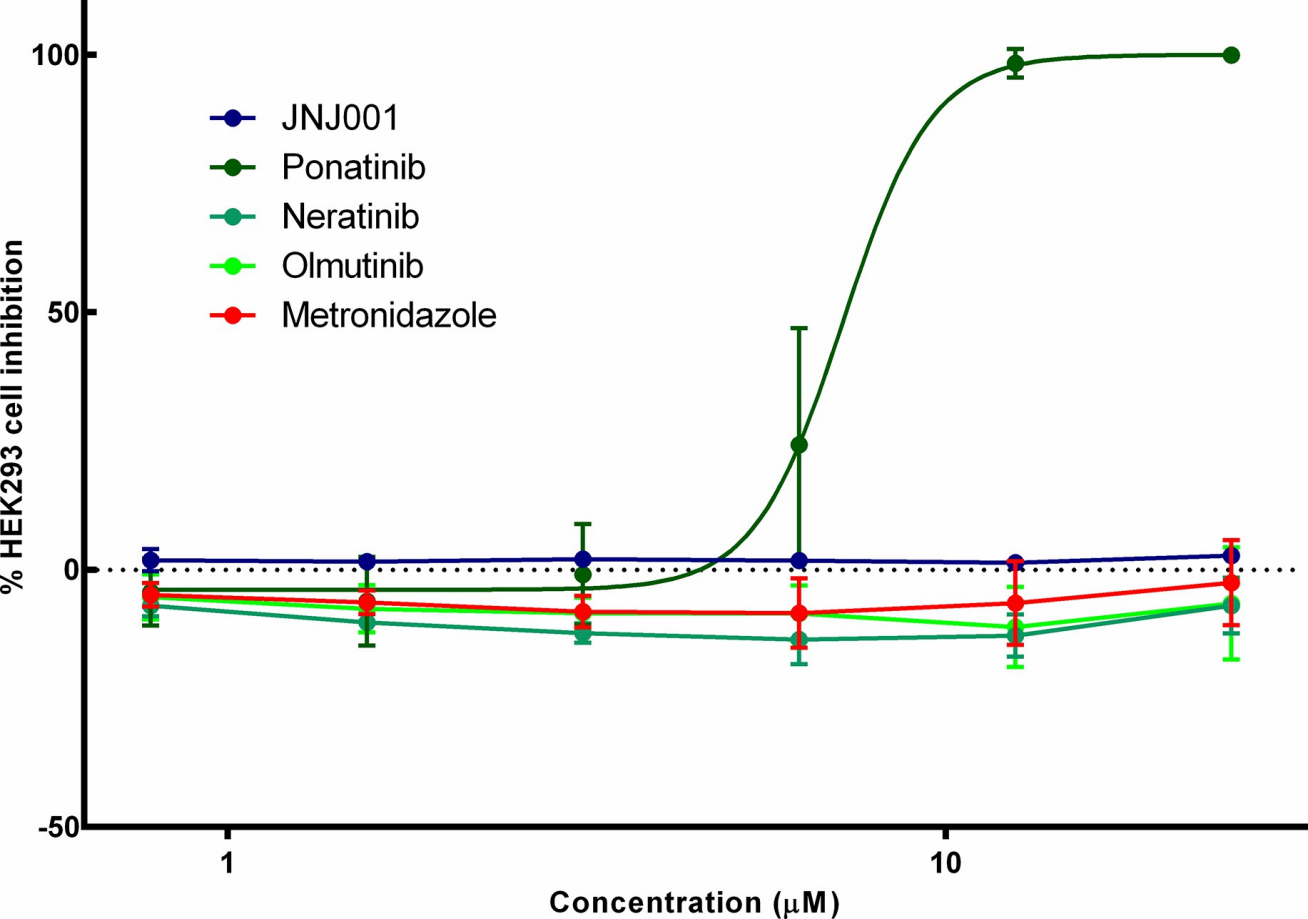
Compound JNJ001 does not inhibit human HEK293 cells. Dose-response curves show percent inhibition of human HEK293 cells compared to compound concentration. Cell viability was measured after 48 hours of exposure to test compounds, and percent inhibition for each compound was calculated relative to positive and negative controls. Compound JNJ001 is represented with a navy-blue line. Metronidazole is represented with a red line. Ponatinib is represented with a green line. Error bars represent standard deviation calculated from four individual measurements of each concentration.

It is a crucial feature of any candidate compound in the drug development process that does not possess toxicity towards human cells, either specifically or as part of a general cytotoxicity. In order to determine whether compound JNJ001 possesses this feature, we tested it for toxicity against human HEK293 cells *in vitro*. In this experiment, human HEK293 cells were cultured and tested in a dose response assay with JNJ001, metronidazole and a selection of antineoplastic kinase inhibitor (AKI) drugs which have previously shown anti-amoebic activity [22]. Of these, only the AKI drug ponatinib produced notable toxicity to the human cells at concentrations up to 25 μM, whereas JNJ001, the other AKI drugs, and metronidazole produced no toxicity at all (Fig 4). These results indicate that JNJ001 is both not specifically toxic to human cells and does not inhibit *E*. *histolytica* due to a generalized cytotoxic mechanism.

### Compound JNJ001 inhibits *E*. *histolytica* as rapidly as metronidazole

**Fig 5:**
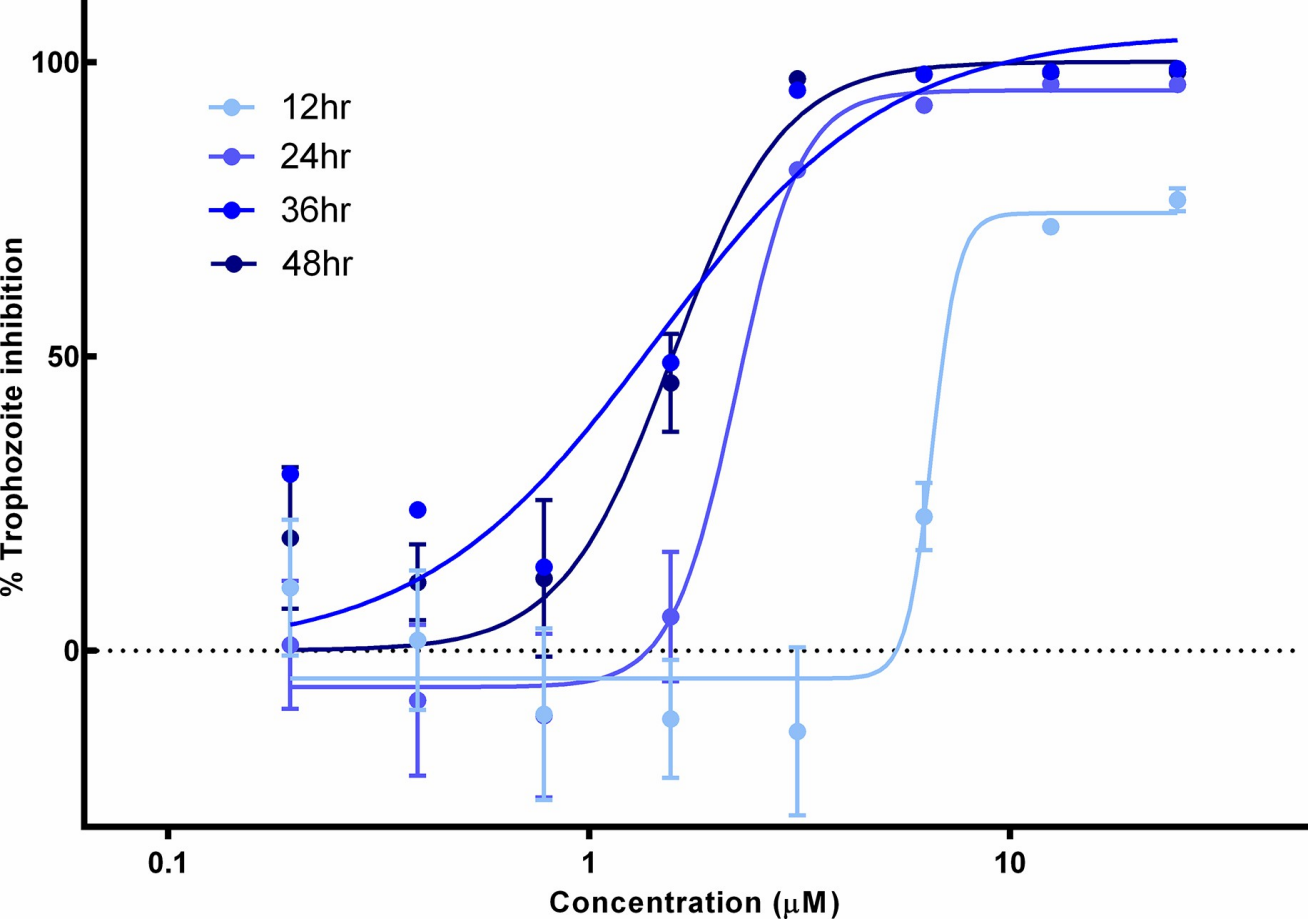
Compound JNJ001 achieves peak anti-amoebic effects within 24–36 hours. Dose-response curves show percent inhibition of *E*. *histolytica* trophozoites compared to compound concentration. Trophozoite cell viability or survival was measured after 12, 24, 36, and 48 hours of exposure to test compounds. Percent inhibition for each data point was calculated relative to average positive and negative control values. Error bars represent standard deviation from three individual measurements at each concentration.

In order to determine the speed with which compound JNJ001 achieves its inhibitory potency against *E*. *histolytica*, we measured and calculated its EC_50_ values at a series of timepoints subsequent to administration, alongside metronidazole for comparison. Cell viability was measured using CellTiter-glo at 12, 24, 36, and 48 hours after the addition of compounds. Both JNJ001 and metronidazole achieved their lowest EC_50_ values between the 24- and 36-hour timepoints (Fig 5), indicating that JNJ001 acts to inhibit *E*. *histolytica* as rapidly as the current standard of care drug.

### Compound inhibits mature *Entamoeba* cysts

**Fig 6:**
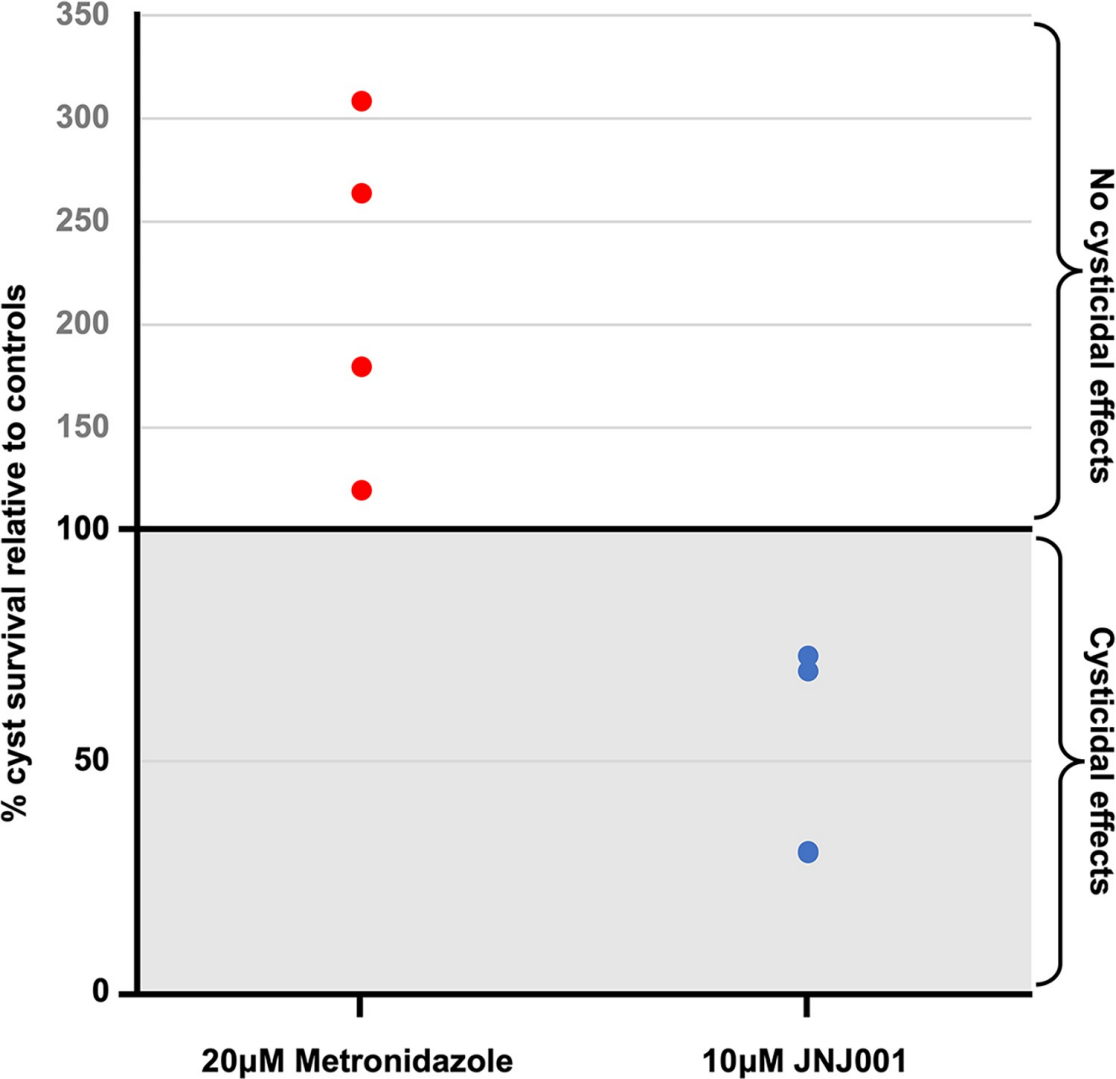
Compound JNJ001 inhibits *E*. *invadens* cysts. Graph representing the survival of luciferase-expressing *E*. *invadens* cysts treated with 10 μM compound JNJ001 (navy blue circle markers) or 20 μM metronidazole (red diamond markers) relative to 0.5DMSO-treated controls. Markers represent individual luciferase readings. Metronidazole data taken from (Sauvey *et al*., 2021) [22].

As *E*. *histolytica* cannot be induced to encyst *in vitro*, we utilized the related parasite, *E*. *invadens*, a well-characterized model system for *Entamoeba* development, to assay for inhibition by compound JNJ001 [36]. Mature (72h) cysts of a transgenic line constitutively expressing luciferase were treated with JNJ001 at 10 μM or 0.5% (v/v) DMSO as a negative control, for 3 days. After treatment, cysts were treated with distilled water for five hours to remove any remaining trophozoites, and luciferase activity was assayed. At 10 μM, JNJ001 was found to significantly reduce of the luciferase signal over 60 percent compared to controls, indicating that it is capable of killing *Entamoeba* cysts (Fig 6). In contrast, 20 μM metronidazole has been previously demonstrated to produce no such inhibition [22]. The results suggest the newly identified compound JNJ0001 has superior activity to metronidazole in killing mature cysts.

### Screen of compounds structurally related to JNJ001 against *E*. *histolytica* trophozoites

**Fig 7:**
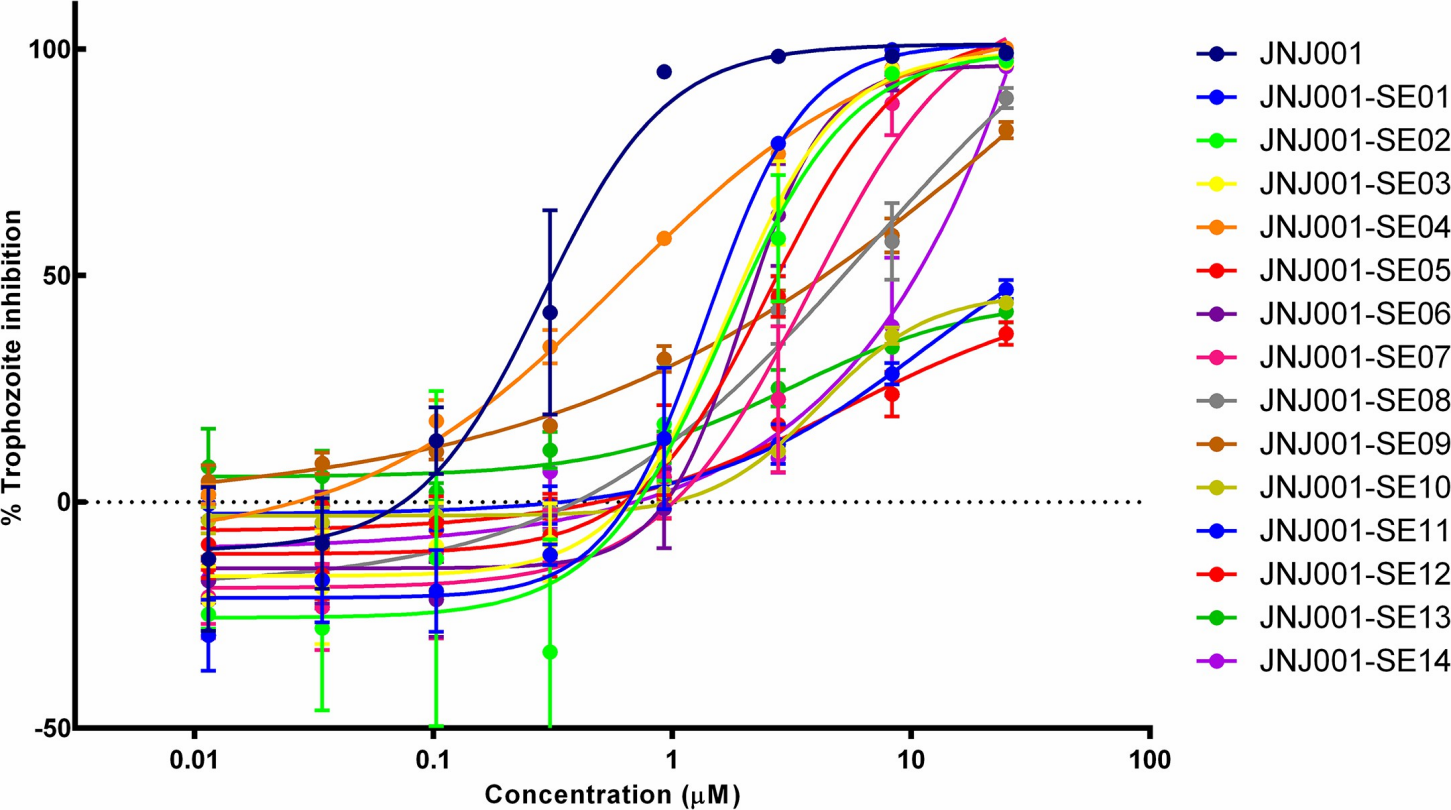
Dose-response assay results for Jump-stARter library JNJ001 series expansion compounds. Dose-response curves show percent inhibition of *E*. *histolytica* trophozoites compared to compound concentration. Trophozoite cell viability or survival was measured after 48 hours of exposure to test compounds, and percent inhibition for each compound was calculated relative to controls. Compound JNJ001 data (shown as a navy-blue line) from Fig 2 is included for comparison. Error bars represent standard deviation calculated from three individual measurements for each concentration.

**Fig 8:**
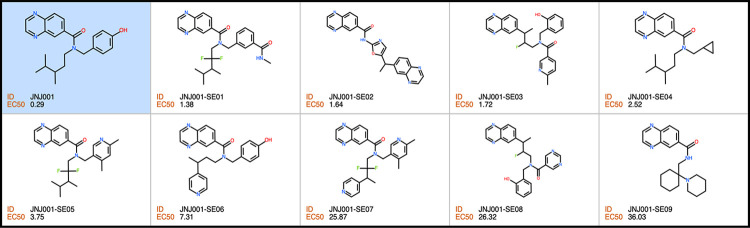
Structures of Jump-stARter library JNJ001 series expansion compounds. Structures, IDs, and measured EC_50_ values of compounds structurally related to JNJ001 (highlighted in blue) found in the Janssen Jump-stARter library.

**Fig 9:**
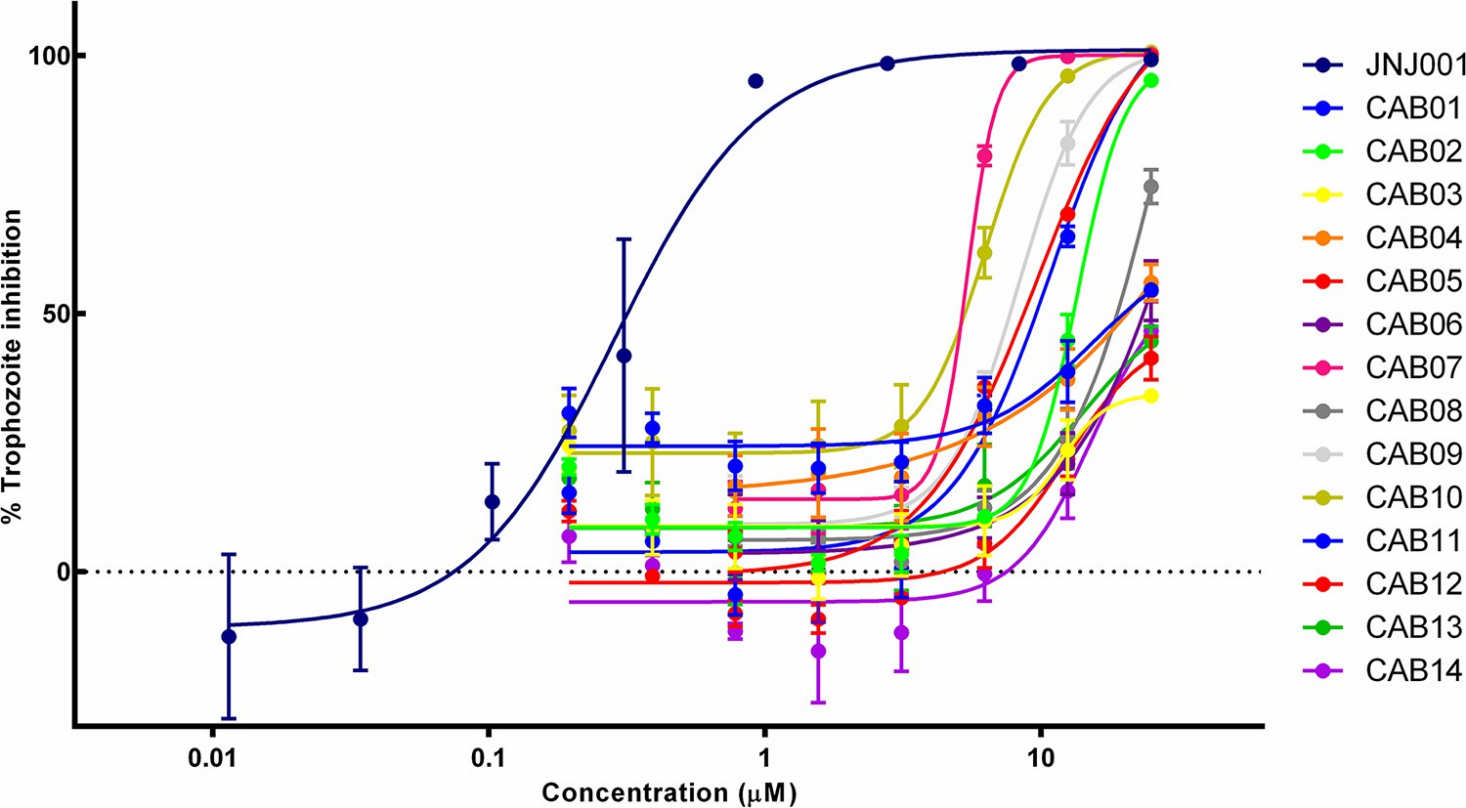
Dose-response assay results for non-Jump-stARter library JNJ001 series expansion compounds. Dose-response curves show percent inhibition of *E*. *histolytica* trophozoites compared to compound concentration. Trophozoite cell viability or survival was measured after 48 hours of exposure to test compounds, and percent inhibition for each compound was calculated relative to controls. Compound JNJ001 data (shown as a navy-blue line) from Fig 2 is included for comparison. Error bars represent standard deviation calculated from three individual measurements for each concentration.

**Fig 10:**
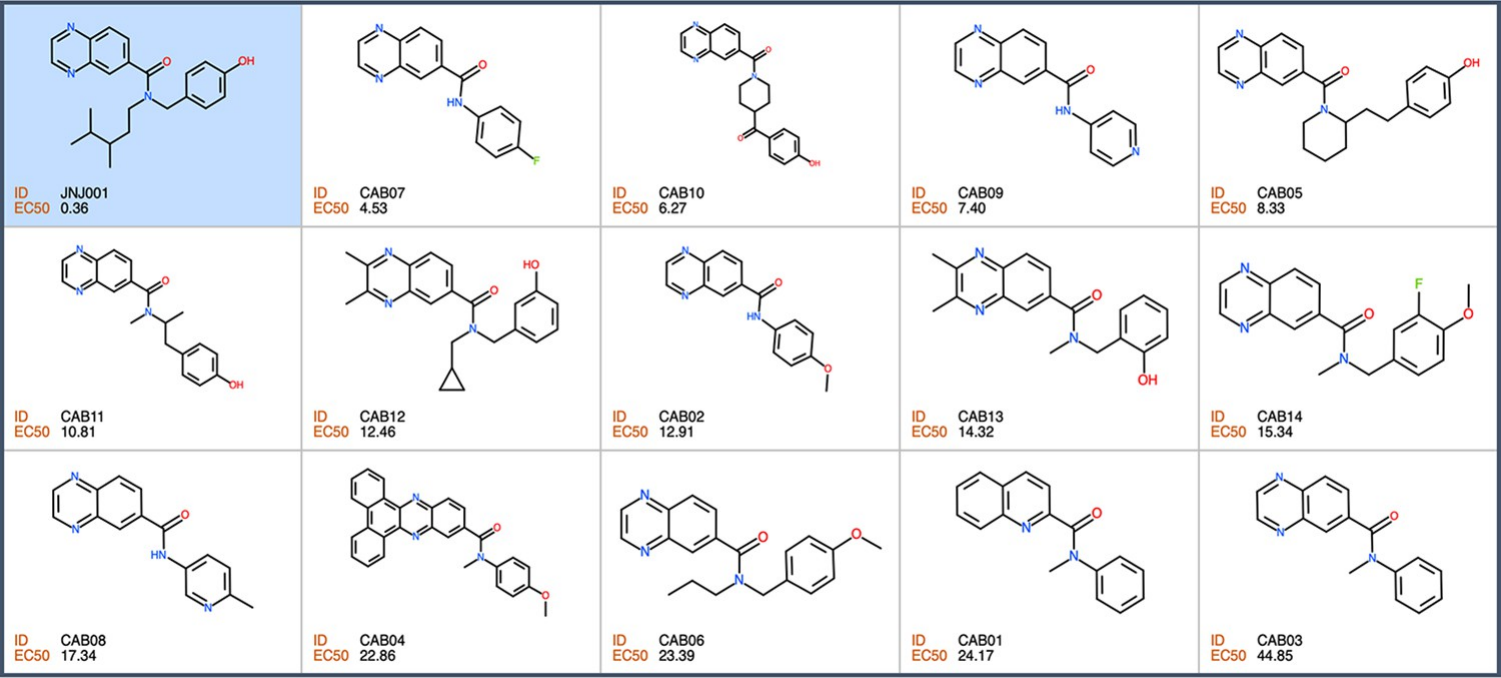
Structures of non-Jump-stARter library JNJ001 series expansion compounds. Structures, IDs, and measured EC_50_ values of additional compounds structurally related to JNJ001 (highlighted in blue).

In order to find additional inhibitors of *E*. *histolytica* based on compound JNJ001, we searched for structurally related compounds in both the hit list from the Jump-stARter library and online chemical vendor catalogs. We began by conducting a structural clustering analysis of the 128 available Jump-stARter library hits from the high-throughput screen. From this clustering analysis we identified 9 structurally similar series expansion compounds (designated as JNJ001-SE01 to JNJ001-SE09) and determined their EC_50_ values using *in vitro* dose-response assays as previously described (Figs 7 and 8). The EC_50_ values for this group of compounds ranged 1.38 μM to 6.57μ M (Table 2). In addition to the biological assay, we determined several key chemical properties for each compounds including molecular weight, predicted solubility (logS), and the ICM ToxScore calculated by the number of unfavorable substructures or substituents present in the molecules. We next conducted substructure- and similarity-based searches of online chemical vendor catalogs and identified 14 purchasable compounds (designated as CAB01-CAB14) similar to JNJ001. These were then purchased and tested to determine their EC_50_ values (Figs 9 and 10). The resulting values ranged from 4.53 μM to 44.85 μM (Table 3). These results together indicate that compound JNJ001 and its structural-related family of small molecules with exhibit suitable chemical and biological properties for further development for anti-amoebic.

**Table 2:** Molecular weight, EC_50_ values against *E*. *histolytica* trophozoites *in vitro*, predicted solubility, lipophobicity, and toxicity of Jump-stARter library JNJ001 series expansion compounds.

Compound ID:	Molecular weight	EC_50_ (μM)	Solubility (LogS)^a^	Lipophobicity (cLogP)^b^	ICM ToxScore ^c^
Metronidazole	171	2−5^22^	-1.17	-0.52	0.45
JNJ001	377	0.29	-4.11	4.76	0
JNJ001-SE01	454	1.38	-4.06	4.23	0
JNJ001-SE02	396	1.64	-3.42	2.28	0
JNJ001-SE03	444	1.72	-3.94	3.90	0.97
JNJ001-SE04	325	1.99	-4.49	4.46	0
JNJ001-SE05	426	2.52	-5.40	4.62	0
JNJ001-SE06	412	2.56	-4.11	3.84	0.22
JNJ001-SE07	461	3.75	-4.61	3.70	0.22
JNJ001-SE08	431	6.57	-2.70	2.68	0.97
JNJ001-SE09	352	7.31	-3.94	3.65	0
JNJ001-SE10		25.87			
JNJ001-SE11		26.32			
JNJ001-SE12		36.03			
JNJ001-SE13		37.01			
JNJ001-SE14		Unstable			

^a^Water solubility (LogS) is the logarithm of the solubility in mol/l calculated using ICM-Pro v3.9 [29].

^b^Lipophobicity (LogP) is the logarithm of the ratio of the compound concentration of in octanol and water calculated using ICM-Pro v3.9 [29].

^c^ICM ToxScore indicates the predicted toxicity of the compound based on chemical structure. ICM ToxScore > 1 indicates the compound contain of unfavourable substructure or substituent. The prediction was performed using ICM-Pro v3.9 [29].

^d^EC_50_ values determined from dose-response assay against *E*. *histolytica* trophozoites *in vitro*.

**Table 3:** Molecular weight, EC_50_ values against *E*. *histolytica* trophozoites *in vitro*, predicted solubility, lipophobicity, and toxicity of non-Jump-stARter library JNJ001 series expansion compounds.

Compound ID:	Molecular weight	EC_50_ (μM)	Solubility (LogS)^a^	Lipophobicity (cLogP)^b^	ICM ToxScore^c^
CAB07	267	4.53	-3.43	2.45	0
CAB10	361	6.27	-2.57	1.73	0
CAB09	250	7.40	-2.13	1.24	0
CAB05	361	8.33	-3.80	2.93	0
CAB11	321	10.81	-2.98	2.26	0
CAB12	361	12.46	-3.78	3.16	0
CAB02	279	12.91	-3.45	2.40	0
CAB13	293	14.32	-2.63	1.74	0.75
CAB14	325	15.34	-2.84	2.18	0
CAB08	264	17.34	-2.86	1.92	0
CAB04	443	22.86	-6.94	5.85	3.2
CAB06	349	23.39	-4.07	3.32	0
CAB01	262	24.17	-4.07	3.07	0
CAB03	263	44.85	-2.90	1.86	0

^a^Water solubility (LogS) is the logarithm of the solubility in mol/l calculated using ICM-Pro v3.9 [29].

^b^Lipophobicity (LogP) is the logarithm of the ratio of the compound concentration of in octanol and water calculated using ICM-Pro v3.9 [29].

^c^ICM ToxScore indicates the predicted toxicity of the compound based on chemical structure. ICM ToxScore > 1 indicates the compound contain of unfavourable substructure or substituent. The prediction was performed using ICM-Pro v3.9 [29].

^d^EC_50_ values determined from dose-response assay against *E*. *histolytica* trophozoites *in vitro*.

### Human cell toxicity assay of series expansion compounds

**Fig 11:**
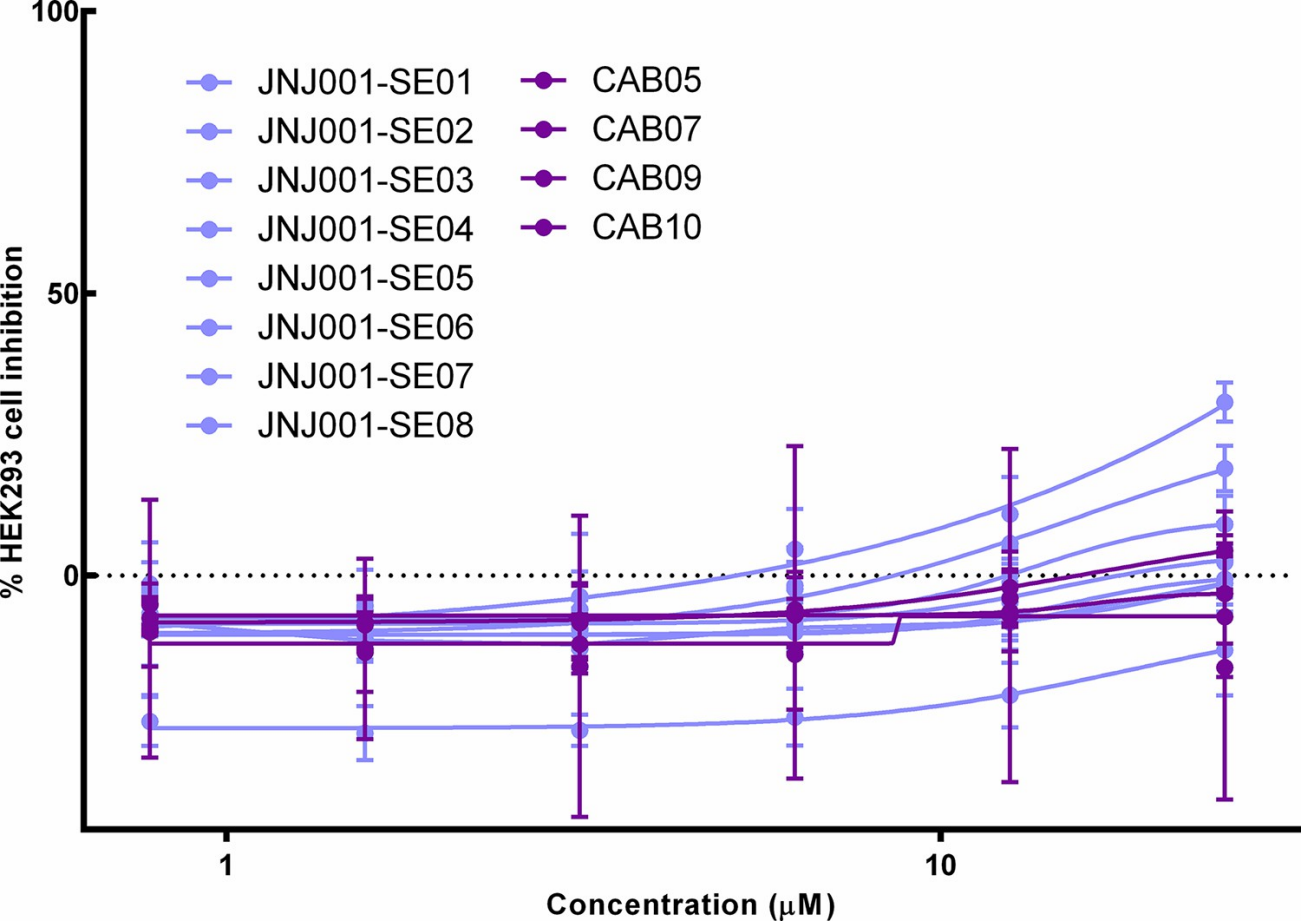
Series expansion compounds do not cause cell death in human HEK293 cells. Dose-response curves show percent inhibition of human HEK293 cells compared to compound concentration. Cell viability was measured after 48 hours of exposure to test compounds, and percent inhibition for each compound was calculated relative to positive and negative controls. Jump-stARter library compounds structurally related to JNJ001 are represented with light blue lines. Purchased compounds structurally related to JNJ001 are represented with pink lines. Error bars represent standard deviation calculated from four individual measurements for each concentration.

In order to determine whether, like JNJ001, the series expansion compounds are cytotoxic to human cells, we tested them in a dose-response assay against cultured human HEK293 cells. Of the compounds tested, none produced notable toxicity to the human cells at concentrations up to 25 μM (Fig 11). These results indicate that the compounds structural related to JNJ001 are not inhibiting *E*. *histolytica* due to a generalized cytotoxicity.

## Discussion

Amoebiasis caused by infection with *E*. *histolytica* remains a significant public health issue in many places throughout the world, despite the longstanding availability of the drug metronidazole as a treatment [3, 4]. Multiple factors may contribute to this, including infrastructural problems in affected areas such as difficulties with water and food sanitation [4]. However, another important factor may be the shortcomings of metronidazole itself. Strong side effects and metronidazole’s inability to kill transmissible cysts complicate the course of treatment and have been suggested to contribute to patient non-compliance and resulting increased disease spread [13, 14]. As such, research into new and better anti-amoebic drugs is ongoing. For much of the history of this research, efforts have focused on inhibiting or disrupting specific cellular processes or targets within the parasite such as redox metabolism or kinase proteins [19]. As an example, several antineoplastic kinase inhibitor drugs were identified as highly potent inhibitors of *E*. *histolytica* [22]. More recently however, there were several studies, have begun to achieve success with non-targeted screens ranging from low-to-high-throughput [20, 21]. In the current study, we continued and expanded upon this approach by conducting a large high-throughput *in vitro* screen against *E*. *histolytica*, resulting in the identification of a promising new family of potent inhibitors of this parasite. The primary objective of this study was to use a high-throughput screening approach to identify novel small-molecule inhibitors of *E*. *histolytica* with high potential for further development as treatments for amoebiasis. Initially, we selected approximately 80,00 screening compounds from Jump-stARter chemical library based on their “drug-like” chemical properties such as appropriate molecular weight, solubility, and lack of known toxic groups. This library was compiled by scientists at Janssen Pharmaceuticals specifically for use in novel drug discovery efforts and has been used in multiple collaborative projects of that nature [25, 26]. The screening library were also selected for structural diversity and clustering into groups of related compounds, thus facilitating expansion of a single hit into a series or family of molecules. The library is also the largest yet screened against *E*. *histolytica*, which further increased the probability of identifying a high potency hit. However, this large library size also represented the next important challenge in our experimental design. Previous screening efforts against *E*. *histolytica* have used a maximum number of wells per assay plate of 96 or 384 [20, 21]. In the case of a library of this size, using plates of those well densities would necessitate using hundreds of them, which would result in a lengthy and complicated screening procedure. In order to avoid this and streamline our process we developed and optimized a screening procedure using 1536-well plates, thus dramatically reducing the overall number of plates required, and hence the time required for screening them. This technical advance also has the additional benefit of opening new possibilities of scale for future screening efforts against *E histolytica*. Once the assay protocol was established, we screened the Jump-stARter library against *E*. *histolytica* trophozoites *in vitro* using a luciferase-based cell viability assay, which effectively measures the adenosine triphosphate (ATP) contained within surviving cells in each reaction volume, which can in turn be compared with average positive and negative control values to obtain a percent inhibition for each compound screened. From the primary screening, compounds exhibited more than 70% inhibition values selected for further biological investigation. We then further tested top-scoring compounds in assays to determine their EC_50_ values, revealing one in particular, compound JNJ001, as highly potent. We calculated the EC_50_ value of this compound as 0.29 μM—more than a full order of magnitude greater than the previously established values for metronidazole (2–5 μM), and hence surpass the current standard of care drug metronidazole in potency [22]. A higher potency could help address several of the drawbacks of metronidazole, such as lowering the dosage required, and potentially reducing adverse effects as a result. The secondary assay direct cell-counting method was conducted to confirm compound as a true positive hit independent of assay type. The trophozoite inhibition values from cell-counting assay were comparable to the results from CellTiter-Glo suggesting that that the signal from luciferase bioluminescent was insignificantly affected by the compounds. The newly identified compound, JNJ001, was tested for the toxicity to human HEK293 cells *in vitro*. We found compound JNJ001 (similarly to metronidazole) to be completely non-toxic at doses up to 25 μM, and far exceeding its EC_50_ value. In addition, the predicted potential toxicity, Tox Score, of JNJ001, from the functional or substituent groups which known to be toxic are zero representing low risk to be a toxic compound which is corresponding to the toxicity investigation result with human HEK293. Its biological activity and the toxicity to human cells indicates the large number of the therapeutic index (TI) and highly effectiveness for killing *Entamoeba*. We also performed a time course assay to compare the compound’s rapidity of action with that of metronidazole and found both it and metronidazole to achieve their final EC_50_ values within 24–36 hours. Together these results confirmed that compound JNJ001 satisfied our criteria which is to equal or surpass metronidazole in other measurable attributes such as rapidity of action and non-toxicity to human cells. These are areas that metronidazole performs well in, and any candidate compound would need to at least match it in order to be considered further. Next, we investigated the ability of compound JNJ001 to inhibit the cysts of *Entamoeba invadens*, a closely related reptilian parasite. In our experiment, compound JNJ001 was found to moderately inhibit *E*. *invadens* cysts, whereas metronidazole did not at all. This moderate cysticidal activity represents a significant advantage of JNJ0001 over metronidazole. These results exhibit JNJ001 as the potential compound for the development of a single molecule that can inhibit both cysts and trophozoites stages, while the current primary treatment for amebiasis relies on multi-drug treatmet [21].

Having successfully identified a compound which fulfilled all the desired criteria, we next sought to expand upon these results and search for additional related molecules with anti-amoebic activity. We used structural clustering to identify several related molecules within the Jump-stARter library. We also used substructure searching to find and obtain additional structural-related molecules from commercial sources. We then took these two sets of series expansion compounds and assayed them for both their EC_50_ against *E*. *histolytica* trophozoites, and their toxicity towards cultured human cells. We found several of the compounds to possess good EC_50_ values, though not as potent as compound JNJ001, and additionally found all of them to possess very low toxicity to human HEK293 cells. These results served as further validation of the anti-amoebic activity of compound JNJ001, confirming that it was not just an isolated phenomenon, but instead can be attributed to a cluster of related molecules with shared activity against this parasite. In addition to biological activity and toxicity assays, the predicted key chemical properties, such as solubility, lipophobicity and ICM ToxScore of all top 24 compounds are satisfied for being drug-like molecules. This discovery also greatly broadened and diversified our set of available anti-amoebic compounds, providing several additional options for further development, some of which may prove to possess even more desirable chemical properties for that process. Taken together the results described in this study indicate the existence of a promising new family of safe, structural-related small molecules with strong anti-amoebic properties against both trophozoites and cysts, and good potential for further development as amoebiasis drugs. Going forward, several areas of further research are now possible. Further studies on the efficacy of these compounds in animal models of *E*. *histolytica* infection would be valuable, though this will be rendered challenging by the difficulties of establishing and maintaining such models [37, 38]. Similarly, safety and tolerability studies in cultured cells, animal models, and humans would be an essential aspect of developing these into clinical treatments. The possibility also exists and can be explored that these compounds may be effective against other related parasitic species. In conclusion, the discovery of these new anti-amoebic compounds represents an exciting new opportunity in the area of amoebiasis research.

## Supporting information

S1 Dataset Fig 1 data. (XLSX)

S2 Dataset 384-well dose-response screen data. (XLSX)

S3 Dataset Fig 2 data. (XLSX)

S4 Dataset Fig 3 data. (XLSX)

S5 Dataset Fig 4 data. (XLSX)

S6 Dataset Fig 5 data. (XLSX)

S7 Dataset Fig 6 data. (XLSX)

S8 Dataset Fig 7 data. (XLSX)

S9 Dataset Table 1 data. (XLSX)

S10 Dataset Fig 11 data. (XLSX)
